# RNA G-quadruplexes (rG4s): genomics and biological functions

**DOI:** 10.1093/nar/gkab187

**Published:** 2021-03-27

**Authors:** Kaixin Lyu, Eugene Yui-Ching Chow, Xi Mou, Ting-Fung Chan, Chun Kit Kwok

**Affiliations:** Department of Chemistry and State Key Laboratory of Marine Pollution, City University of Hong Kong, Kowloon Tong, Hong Kong SAR, China; School of Life Sciences, and State Key Laboratory of Agrobiotechnology, The Chinese University of Hong Kong, Shatin, Hong Kong SAR, China; Department of Chemistry and State Key Laboratory of Marine Pollution, City University of Hong Kong, Kowloon Tong, Hong Kong SAR, China; School of Life Sciences, and State Key Laboratory of Agrobiotechnology, The Chinese University of Hong Kong, Shatin, Hong Kong SAR, China; Department of Chemistry and State Key Laboratory of Marine Pollution, City University of Hong Kong, Kowloon Tong, Hong Kong SAR, China; Shenzhen Research Institute of City University of Hong Kong, Shenzhen, China

## Abstract

G-quadruplexes (G4s) are non-classical DNA or RNA secondary structures that have been first observed decades ago. Over the years, these four-stranded structural motifs have been demonstrated to have significant regulatory roles in diverse biological processes, but challenges remain in detecting them globally and reliably. Compared to DNA G4s (dG4s), the study of RNA G4s (rG4s) has received less attention until recently. In this review, we will summarize the innovative high-throughput methods recently developed to detect rG4s on a transcriptome-wide scale, highlight the many novel and important functions of rG4 being discovered *in vivo* across the tree of life, and discuss the key biological questions to be addressed in the near future.

## INTRODUCTION

**Figure 1.**
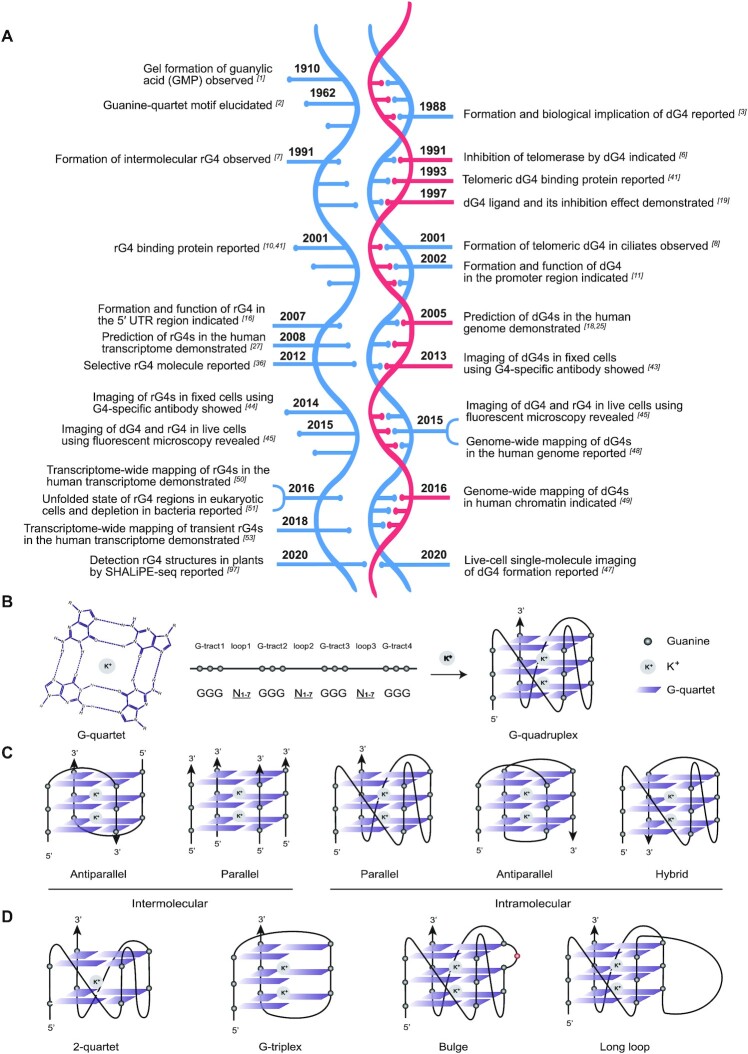
Milestones in G-quadruplex (G4) and the structural diversity of G4s. (**A**) Key discoveries and developments in rG4 (left) and dG4 (right). This is a huge topic and we apologize for any omission. (**B**) Structure of a G-quartet, and canonical G4 with G3N1-7 sequence consensus. Potassium ion (K^+^) stabilizes the G4. (**C**) Representative topologies of intra- and inter-molecular G4s. (**D**) Representative type of non-canonical G4s.

The origin of research into the G-quadruplex (G4) can be traced back more than a century to 1910, when Bang reported the formation of a gel structure from an RNA monomer, guanosine monophosphate, at high concentration (1) (Figure 1A). Around 50 years later, this peculiar structure was solved by Gellert and colleagues using X-ray diffraction, and the G-quartet motif was proposed (2) (Figure 1B). In a G-quartet, each guanine base serves as both a hydrogen bond donor and a hydrogen bond acceptor in two base pairs to form a planar structure, and two or more G-quartets can stack on top of each other to form a G4 (Figure 1B). The first demonstration of the formation and biological significance of the DNA G-quadruplex (dG4) structure, at the telomere region and immunoglobulin switch regions, was reported by Sen and Gilbert in 1988 (Figure 1A) (3). Since then, many initial structural and biochemical studies have shown that G-rich DNA or RNA sequences with G4-forming potential can fold into dG4s and RNA G-quadruplex (rG4) structures (4–9), and play vital roles in various cellular functions (10–17). The potential to form G4s can be predicted from the primary sequences. Canonical putative G-quadruplex sequences (PQSs) contain four runs of three consecutive guanines separated by three loops, with the loop length ranging from one to seven nucleotides (GGGN_1-7_GGGN_1-7_GGGN_1-7_GGG), or G_3_N_1-7_ for short (Figure 1B) (18). The formation of G4s is stabilized by cations such as K^+^, which can fit into the space between two quartets (Figure 1B). The G4-stabilizing effects of monovalent ions decrease in the following order: K^+^ > Na^+^ > Li^+^ (14). Similarly, small molecules that target G4 can stabilize the structure, and the first G4-targeting compound was developed to inhibit the telomerase-mediated telomere extension (19). G4s can be classified as intermolecular and intramolecular structures (Figure 1C) (20). Intermolecular G4s are formed by two or four separate strands of DNA or RNA, whereas intramolecular G4s are formed by a single DNA or RNA strand (Figure 1C). G4s can also be classified topologically as parallel, anti-parallel or hybrid (Figure 1C). rG4s prefer to form in the parallel topology due to the anti-conformation of glycosidic bonds in ribonucleosides, and except for a few special cases (21–23), most of the reported rG4s to date adopt a parallel topology. In contrast, dG4s can adopt parallel, antiparallel or hybrid topologies and switch from one to another, depending on physiochemical conditions and sequence properties (24). Based on systematic computational analyses of the human genome, over 375 000 canonical PQSs were reported for the first time in 2005 (Figure 1A) (18,25). PQSs are now known to be prevalent and non-random, localizing in functionally important regions of the genome, such as in telomeres (5), promoters regions (26) and untranslated regions (UTRs) of messenger RNAs (mRNAs) (27). Moreover, many PQSs are highly conserved among mammalian species (28), which further implies their biological functionality.

Although dG4s and rG4s share the basic structural motif—the G-quartet—they are distinct. First, rG4s are more compact and thermally stable than their DNA counterparts (29–32) due to the 2′-hydroxyl group in the ribose sugar and the networks of water-mediated contacts within the grooves of RNA (33). This compactness allows more intramolecular interactions within rG4s. Second, dG4s and rG4s show distinct preferences in binding specificity with cations (34) and ligands (35,36). K^+^ adequately stabilizes both types of G4, whereas Na^+^ sometimes has a strong effect only on dG4s but not rG4s (37). Due to the 2′-hydroxyl groups in RNA and their effects on the groove and loop widths, some G4 ligands interact less effectively with rG4s than with dG4s (35). Third, in eukaryotes, DNA mostly exists in a classical double-stranded helix conformation in the nucleus, whereas RNA is single-stranded in nature and located in both the nucleus and cytoplasm. Therefore, RNA in general has more freedom to fold into diverse secondary structures including rG4s, and has access to a great diversity of binding partners, such as proteins, in different cellular compartments (38,39). The first dG4-binding protein, the β subunit of the Oxytricha telomere-binding protein, was identified in 1993 (40), but it was until 2001 that the first rG4-binding protein, fragile X mental retardation protein (FMRP), was reported (Figure 1A) (10,41). Over the last two decades, several of G4-binding proteins have been identified to interact with rG4 structures and play regulatory roles by folding or unfolding the G4 structures or recruiting more binding partners (38,42). In 2013–2014, G4s were found to exist in fixed human cells in studies using G4-specific antibodies, BG4 (Figure 1A) (43,44). Similarly, G4-specific turn-on probes, and more recently a single-molecule fluorescence imaging probe (SiR-PyPDS), were developed to detect G4s in live cells (45–47). Shortly after the report of genome-wide mapping of dG4s in genomic DNA *in vitro* and chromatin *in situ* (48,49), transcriptome-wide mapping of rG4s in the human transcriptome was reported in 2016 (Figure 1A) (50,51). In these works, besides canonical G4s, non-canonical G4s such as two-quartet G4s, G-triplexes, bulged G4s and long-loop G4s were found to be prevalent (Figure 1D), but the *in vivo* folding status of many of these G4s, especially rG4s, detected *in vitro* was in question (50–52). Several studies in the last few years have suggested a dynamic and transient behaviour of rG4s *in vivo* (53,54), and many new rG4s with critical functions have been revealed not only in mRNAs, but also in non-coding RNAs (ncRNAs) (55,56).

Compared with dG4s, rG4s had received less attention until recently. With the continual emergence of data on the innovative transcriptome-wide detection and novel cellular roles of rG4s, this review aims to provide a comprehensive summary and critical analysis of the latest advancements in the rG4 field, and particularly on rG4 transcriptomics and functions in different biological systems, which are currently missing in recent reviews. We summarize the high-throughput techniques developed to map rG4s on a global scale. We also highlight the crucial functions of rG4s and their mechanisms elucidated in mammalian species, viruses, plants and bacteria. In each respective section, we also discuss current challenges and future perspectives to address the open questions in the rG4 field.

### rG4 genomics in its development stage—from genome- to transcriptome-wide rG4 detection

Investigation of rG4 biology from a genomic approach requires knowledge of many, if not the entirety of rG4 structures harbored in the genome of an organism-of-interest. A comprehensive and accurate list of rG4s would support the inference of associations between rG4s and other genomic elements, where such information holds promise to reveal the general functional significance of rG4s in biological systems. Unfortunately, because most of the classical experimental rG4 detection methods are low-throughput, the lack of a suitable genomic-wide rG4 profiling method has been the major obstacle in adopting a genomics approach in rG4 research (52).

To circumvent that limitation, pioneering researchers opted for an alternative approach by first computationally predicting PQSs in a genome-of-interest, then selecting the subset of sequences overlapping with transcribed gene regions as the input of rG4 genomic studies. Therefore, it is common to find existing rG4 genomic studies based predominantly on computationally predicted rG4 structures (57,58). Logically, the fidelity of these studies depended on the performance of the prediction methods in capturing the biophysical phenomena underlying rG4 structures. To offer a clearer picture of the theoretical basis of rG4 genomic studies, we first revisit the major methods and results that contributed to the bulk of our existing knowledge on rG4s, which was obtained through the iterations of

**Figure 2.**
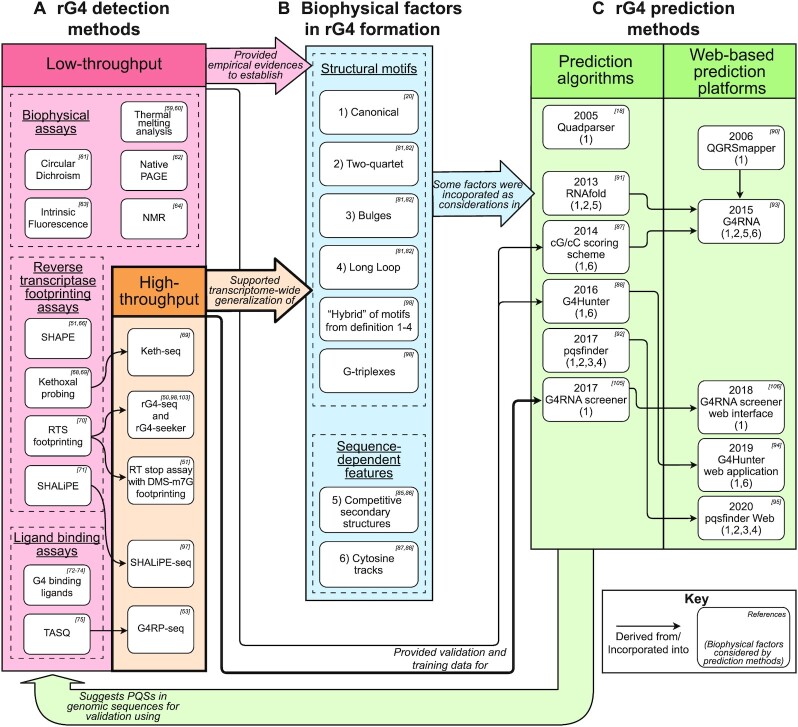
An overview on the current paradigm of rG4 genomic research. (**A**) rG4 detection methods identify rG4 structures in transcribed genomic sequences experimentally. (**B**) Biophysical factors in rG4 formation are generalized from the repository of experimentally confirmed rG4 structures. (**C**) rG4 prediction methods provide the genome-wide screening of PQSs, through identifying genomic sub-sequences that fulfill a subset of biophysical factors in rG4 formation.

1. Low-throughput experimental validation of individual PQSs to identify rG4-forming sequences (Figure 2A)
2. Generalization of biophysical rules governing the rG4 formation propensity from experimental evidence (Figure 2B)
3. Establishment of new rG4 prediction methods built upon the generalized findings and propensity rules (Figure 2C)

In an ideal situation, rG4 prediction methods would identify rG4 structures from input transcript sequences reliably, thus alleviating the need for experimental validation. However, all existing rG4 prediction methods have limited predictive power and can only identify PQSs, whereas their rG4 formation capability remains uncertain and requires confirmation using low-throughput rG4 detection assays, e.g. circular dichroism. The requirement for low-throughput assays then bottlenecks the discovery rate of new rG4 structures, which further limits the number of rG4s available for use to generalize biophysical factors in rG4 formation, and thus degrades the capabilities of the factors to fully describe the rG4 formation propensity. As a result, rG4 prediction methods incorporating these factors can only achieve mediocre prediction power, thus effectively leading to a cyclic problem that ultimately prevents reliable identification of all rG4s in genomes.

To overcome this problem, significant efforts have been invested to develop high-throughput experimental rG4 detection methods that profile rG4s at a transcriptome-wide scope, without requiring PQS input from rG4 prediction methods. These novel methods share a common approach of coupling classical rG4 detection chemistries with RNA sequencing (RNA-seq) technology, which has greatly enhanced their throughput from one to hundreds/thousands of rG4s per experiment. Importantly, the structural features of rG4s detected with high-throughput methods are coherent with our understanding of their biophysics and the results from their low-throughput counterparts. In the second part of this section, we review several high-throughput rG4 detection methods available to date, summarize their capabilities and notable features and discuss their general significance in rG4 research.

Nevertheless, the emergence of high-throughput rG4 detection methods has introduced not only new opportunities in rG4 genomic research, but also new challenges. These include their high sample requirements and sensitivity to transcript abundance in RNA samples, as well as the large number of rG4 detections, which can be overwhelming for human interpretation. In the last part of this section, we discuss our perspectives on how the challenges imposed by high-throughput methods may be overcome, and how their findings could be utilized to answer pending questions in different facets of rG4 research.

### Low-throughput rG4 detection methods

The general objective of rG4 detection methods is to determine the presence/absence of rG4 structures within an RNA region-of-interest. Low-throughput assays are most applicable to genomic regions that have already been suggested to harbour PQSs, but whose rG4 formation capabilities are yet to be experimentally confirmed. These methods typically evaluate one short RNA sequence (<100 nt) at a time. The rG4 detection methods developed to date can be broadly classified into three major types based on their working principles (Figure 2A):

-**Biophysical assays** are the earliest type of assay available for detecting rG4s. These techniques either evaluate changes in biophysical metrics, such as RNA melting temperature (59,60), spectrometric absorbances (61), migration velocity in acrylamide gel (62) or intrinsic fluorescence (63), which vary between the unfolded and folded forms of rG4s; or directly determine the unique imino proton chemical shifts of G-quartet base pairing or the 3D structure of a PQS (64). Because these metrics are unique to rG4 motifs, these assays remain the gold standards for rG4 detection (65). Nonetheless, biophysical assays might not fully reflect the influence of flanking sequences and *in viv*o environments in rG4 formation, as they typically measure rG4 folding of homogeneous and short RNA oligonucleotides.
-**Reverse transcriptase (RT) footprinting assays** are a genre of techniques that exploit the intrinsic behaviour of reverse transcriptases, where cDNA synthesis can be blocked by covalently modified RNA residues (66) or thermostable RNA structures (67) on the template RNA. The footprints of blocked cDNA synthesis at some positions on a transcript, also known as a reverse transcriptase stalling (RTS) event, can then be detected by techniques such as denaturing polyacrylamide gel electrophoresis (PAGE).
  The major innovation underpinning the detection of RNA structure by RT footprinting involves the development of chemicals that selectively modify unstructured nucleotide residues. Structured and unstructured regions on RNA molecules can then be identified based on the differences in chemical modification rate, which are indicated by the observed frequency of RTS events. Methods that see applications in rG4 detection include selective 2′-hydroxyl acylation analysed by primer extension (SHAPE) (51,66) and kethoxal footprinting (68,69). Although these methods are compatible with capturing *in vivo* RNA structures, they are unable to clearly differentiate between double-stranded RNA (dsRNA) and rG4s, and could be ambiguous in situations where an rG4 competes with an alternative RNA structure, e.g. dsRNA, to form at locus-of-interest.
  An alternative approach to detect rG4 specifically with RT footprinting involves incorporating an *in vitro* rG4 folding step: when supplied with an rG4-stabilizing buffer solution of physiologically relevant K^+^ concentration and optionally in the presence of rG4-stabilizing ligands, rG4 structures can fold stably to block reverse transcription at their 3′ end. The reaction is then repeated in a non-rG4-stabilizing buffer, typically of Li^+^ concentration of the same ionic strength, which prevents rG4s from folding. RTS events that are specific to rG4-stabilizing conditions can then be identified to infer the presence of rG4 structures. Because the formation of dsRNA structures is not known to be sensitive to monovalent cations and rG4 ligands, the method can effectively differentiate rG4s from dsRNA (70). However, the method is unable to capture the *in vivo* folding status of rG4s as native RNA structures are difficult to preserve during cell lysis and RNA extraction procedures. Moreover, since an rG4 structure typically only leaves RT footprint at its 3′ end: if 5 or more G-tracts are present in adjacent on a transcript, it may be difficult to determine the sequence span of a rG4 motif utilizing only footprinting information.
  To overcome these limitations, variations of the technique have been further developed by preserving *in vivo* structural information with chemical modifications. One such method is known as selective 2′-hydroxyl acylation with lithium ion-based primer extension (SHALiPE), which compares the *in vivo* and *in vitro* SHALiPE data of PQSs to identify rG4s that can fold in both conditions from their characteristic SHALiPE footprints (71). An alternative method described by Guo *et al.* utilizes dimethylsulphate (DMS) to methylate guanine tracts of unfolded *in vivo* rG4s at N7 positions, where N7-methyl-guanine (m7G) does not support rG4 refolding under *in vitro* rG4-stabilizing conditions (51). In the respective methods, the additional SHALiPE and m7G information can both reveal the *in vivo* folding status of rG4 structures and cover the entire span of rG4 motif.
  In general, because a sequence-specific reverse transcription primer can be used in PAGE, RT footprinting assays are typically compatible with native RNA extracted from biological samples. In contrast, these methods may face ambiguity when the targeted footprint is not unique to rG4s.
-**Ligand-binding assays** rely on chemical ligands that possess a binding specificity to G4s. In general, many of these ligands are approximately planar in shape and interact with G4s by approaching from the anterior/posterior of the structure by stacking (72). The ligand–G4 interaction can then be detected by ligand-induced fluorescence enhancement in spectroscopy (73), engineering of the ligand to carry fluorophores for imaging (74,75) or attachment of molecular tags such as biotins for pull-down (53). These applications allow the spatial detection of rG4s and the enrichment of rG4-containing RNAs for further analysis, which are unique advantages of ligand-binding assays. However, some G4 ligands can induce and stabilize G4s (76) that do not naturally occur under physiological conditions (77,78). Moreover, given the diversity of rG4 structural conformations, a single type of ligand may not be capable of ubiquitously binding and detecting all rG4s in the genome.

### Biophysical rules governing dG4 and rG4 formation propensity

It has been established that the presence of an rG4-forming sequence of four G-tracts spaced by three connective loops is not always sufficient to infer rG4 folding and formation. Other biophysical factors, such as the lengths of G-tracts/loops and the nucleotide sequence context, have been hypothesized and later experimentally validated to affect the propensity for rG4 formation (79–82). Therefore, there have been numerous attempts to deduce a generalized understanding of how these biophysical factors influence G4 formation. Most of the generalizations were established based on human interpretation of common features (e.g. length and nucleotide compositions) among a few hundreds of rG4s identified with low-throughput detection assays, followed by experiments that tested for the boundary cases. These features can be classified as structural motifs and sequence-dependent features (Figure 2B):

-**Structural motifs** are definitions of the range of G-tracts and loop lengths that are most likely to support G4 formation. They are generally assumed to be interoperable between dG4s and rG4s.
  The most primitive structural motif is referred to as the canonical motif, which refers to rG4s with G-tract lengths of ≥3 nt and loop lengths of 1–7 nt (20). Meanwhile, G4-forming sequences that deviate from the canonical motif definition have been also discovered (83,84). At the expense of reduced thermostability, these G4s tolerate additional structural imperfections in having only two layers of G-quartet instead of three, significantly longer connecting loops, or bulges in one or more G-tracts. Eventually, two studies in 2013 systematically evaluated the landscape of these imperfections using dG4s (82) or rG4s (81) as models, generalized the correlation between their sizes and negative effect on the stability of G4 structures, and offered new sets of non-canonical structural motifs now known as ‘long-loop’, ‘bulged’ and ‘two-quartet’ to describe these G4s (81,82).

-**Sequence-dependent features** refer to the biophysical factors that exhibit variable influences of G4 formation under different nucleotide sequence contexts. To date, competitive secondary structures and cytosine tracks are the only two factors that have been systemically characterized and are believed to apply to rG4s.
  The competitive secondary structure factor expresses the likelihood of a quadruplex sequence to fold into a dsRNA structure instead of a quadruplex structure under favourable free energy conditions (85), for instance in cases where the flanking sequence and the quadruplex sequence are highly complementary. The phenomenon was first experimentally verified in 2012, where a hairpin-to-rG4 conformation transition of an RNA was achieved by modifying the concentration of rG4-stabilizing cations in buffer (86).
  The cytosine track factor expresses the likelihood of cytosine residues within or adjacent to quadruplex sequences to sequester critical guanines (e.g. constituents of the G-quartet) via Watson–Crick base pairing, and thus inhibit rG4 formation. The hypothesis was first experimentally proven and established as a scoring system in a 2014 study (87). The relative ratio between guanine tracks and cytosine tracks near a quadruplex sequence was then shown capable of forecasting the rG4 formation propensity in 2016 (88).

### dG4 and rG4 prediction methods

In outline, G4 prediction methods utilize our understanding of G4 formation propensity to conduct searches for PQSs within a specific nucleotide sequence or in the entire genome, and optionally report prediction score derived from biophysical factors that influence the G4 formation propensity (52,89). Except for methods that are considered rG4-specific, G4 prediction results are generally assumed to be applicable to both dG4s and rG4s. It is worth noting that not all biophysical factors are considered by some prediction methods (Figure 2C).

Meanwhile, G4 prediction methods are commonly classified as either prediction algorithms or Web-based prediction platforms based on their user interfaces. Prediction algorithms are typically mathematical formulas or computer software that implement the PQSs screening logics, whereas web-based prediction platforms incorporating these algorithms serve as a more accessible interface for researchers (Figure 2C). At the researcher's discretion, the complete list of predicted PQSs can then be used as input for a genomic study, or a few PQSs overlapping biological regions-of-interest (e.g. a gene-of-interest, regulatory elements) can be handpicked for experimental validations and downstream structural and functional investigations.

The earliest iterations of G4 prediction methods are primitive in nature, and only search for PQSs matching specified G-tract lengths and loop lengths in an input nucleotide sequence. The canonical structural motif was first incorporated as the basis of prediction in two 2005 studies (18,25), where one of the studies consolidated their *quadparser* algorithm into a command-line program (18). *QGRSmapper*, which was published shortly after the two studies, brought improvements including a web interface and options for users to customize the G-tract lengths and loop lengths to search for (90). The simplicity of *QGRSmapper*’s design has enabled it to remain as a popular tool for rG4 research to this day. However, although *QGRSmapper* offers a ‘G-score’ to evaluate the likelihood for PQSs to form a stable G4, the correlation between G-score and rG4 formation propensity has not been formally certified with empirical data.

Following the establishment of several rG4-specific biophysical factors in the early 2010s, the next iteration of prediction methods newly incorporated non-canonical rG4 structural motifs for PQS searching, and/or sequence-dependent features for deriving metrics that reflect the likelihood of rG4 formation. They included *RNAfold* (91), the *cG/cC scoring scheme* that considered guanine/cytosine tracts (87), *G4Hunter* (88) and *pqsfinder* (92). Notably, the *cG/cC scoring scheme* and *G4Hunter* also leveraged the list of experimentally verified rG4s to validate their algorithms (Figure 2A, C), suggesting both rG4 detections and biophysical factors can contribute to the formulation of prediction methods. Later, the functionalities of *QGRSmapper*, *RNAfold* and the *cG/cC scoring scheme* were integrated into a web-based tool named *G4RNA* (93). Meanwhile, the authors of both *G4Hunter* and *pqsfinder* offered web interfaces for their algorithms in 2019 (94) and 2020 (95), respectively. The timeline of development, dependencies and the biophysical factors that these prediction algorithms and web-based prediction platforms considered is illustrated in Figure 2C. In comparison to *QGRSmapper*/*quadparser*, these newer methods can capture non-canonical PQSs more comprehensively and offer more metrics/scores that illustrate the rG4 formation propensity of PQSs. They also leverage experimentally confirmed rG4s to establish and/or benchmark the prediction methods. Nevertheless, although the new metrics/scores are correlated with the rG4 formation propensity, these prediction methods do not utilize such information to differentiate between PQSs that fold into rG4 structures and those that do not.

Most importantly, all of the above-reviewed G4/rG4 prediction tools assume that users will experimentally validate the predicted PQSs for rG4 formation ability before applying them as input for rG4 biological studies, as they do not offer predictions of the rG4 folding/non-folding status of PQSs. Therefore, for rG4 genomics studies that directly utilize PQS prediction results without further validation, their findings must be interpreted with a caveat that the list of PQSs might contain a significant proportion of false positives.

In this section, we focused on discussing the subset of methods that are either foundational to rG4 research or have considered rG4-specific factors. A recent compresensive review by Lombardi *et al.* (89) provides additional background of diverse G4 prediction methods regarding their implementations and performances.

### High-throughput rG4 detection methods

**Figure 3.**
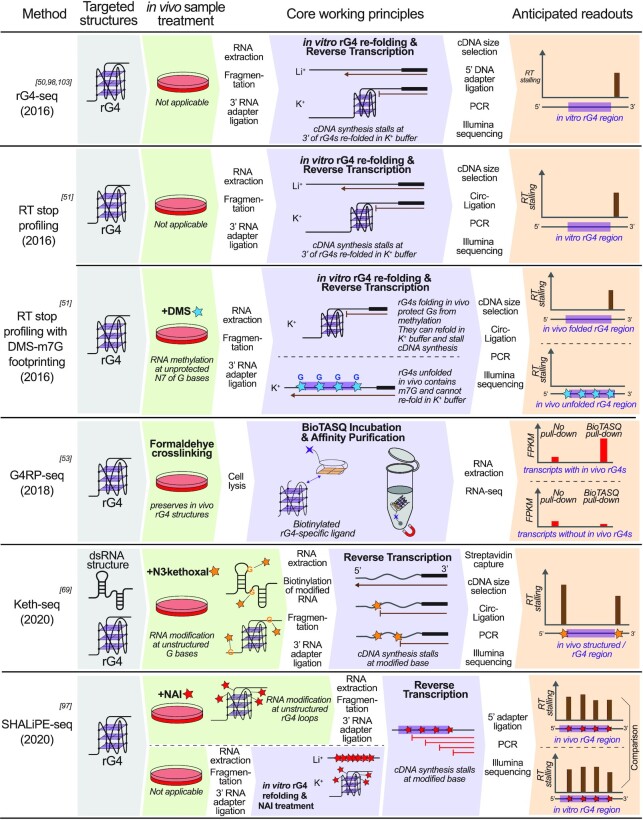
Summary of high-throughput rG4 detection methods.

High-throughput RNA structural determination methods first emerged in the early 2010s following the maturation and popularity of RNA-seq technology. High-throughput assays are a development of their low-throughput counterparts that can evaluate a heterogeneous mixture of RNA sequences in parallel. They are usually applicable to a transcriptome-wide screening of rG4s without requiring PQS predictions *a priori*. Here we review a subset of these methods that are suitable for rG4 detection (Figure 3):

-**Non-rG4-specific RT footprinting methods**
  Keth-seq is currently the only high-throughput RNA structural determination method that can claim a detection of rG4s alongside dsRNA regions (69). It was applied to map *in vivo* RNA structures including rG4s in human and mouse by employing cell models (69). The method works similarly to its low-throughput counterpart by using N^3^-kethoxal to label unstructured guanine bases and induce RTS. The RT footprints are then captured via sequencing to infer the *in vivo* folding/non-folding status of rG4s based on the absence/presence of footprints at the G-tracts. Given an input of PQSs, Keth-seq is effective in determining their respective *in vivo* rG4 folding status by evaluating whether the putative G-tracts are structured and protected from kethoxal modification; however, the possibility that the PQSs will form a dsRNA structure must also be accounted for. In contrast, Keth-seq is less suitable for identifying rG4s that fall outside the predicted list of PQSs, for instance novel non-canonical rG4s based on kethoxal footprints, as these footprints might originate from dsRNA regions instead.

-**rG4-specific, RT footprinting methods**
  The rG4-seq method (50) and the RT stop profiling method with DMS-m^7^G footprinting (51) were the first methods for specific rG4 detection by RT footprinting. rG4-seq was first applied to map *in vitro* rG4 in human by employing a cell model (50) and later to plants and bacteria (96,97); while the RT stop profiling method was applied to map *in vitro* and *in vivo* rG4 in yeast and *Escherichia coli* cells (51). The two methods share an *in vitro* rG4 refolding step, which allows them to determine the 3′ end of *in vitro* rG4s in a similar fashion to their PAGE-based low-throughput counterpart. The RT stop profiling method differs from the rG4-seq method by the incorporation of an additional *in vivo* DMS treatment step, which methylates guanine residues in unfolded rG4s at their N^7^ position. Because m^7^G does not support G-tetrad and G4 formation, the phenomenon can be exploited to infer the *in vivo* folding status of rG4s. Later, in 2020, the SHALiPE-seq method, which was derived from its low-throughput counterpart SHALiPE, was developed and applied to map *in vivo* rG4s in *Arabidopsis thaliana (A. thaliana)* and *Oryza sativa* (*O. sativa*, or Rice) (97). In SHALiPE-seq, *in vivo* rG4s and dsRNA are probed using SHAPE chemistry coupled with sequencing. Simultaneously, *in vitro* SHALiPE probing is conducted in both rG4-stabilizing and rG4-non-stabilizing conditions to specifically identify *in vitro* rG4s. Finally, by combining and comparing the three sets of data, rG4s that fold both *in vivo* and *in vitro* can be identified from their characteristic SHALiPE footprints.

The unique advantage of RT footprinting assays with rG4 refolding lies in their intrinsic rG4-specificity, which allows them to work without an input of known PQSs. Combined with tailored bioinformatic software such as rG4-seeker, which was engineered to work with rG4-seq, an *ab initio* rG4 identification can be achieved by detecting all RTS sites that occur only under rG4-stabilizing conditions. In contrast, this genre of assays must rely on external information, such as DMS-m^7^G footprinting or SHALiPE, to determine the *in vivo* folding status of rG4s. Consequently, *in vivo* rG4s that do not refold under *in vitro* conditions cannot be detected and profiled using these assays.

-**rG4-specific, ligand-binding methods**
  G4RP-seq is currently the only high-throughput rG4 detection method that takes a ligand-binding approach in cells (53). The method uses a biotinylated G4-specific ligand/probe, BioTASQ, to achieve affinity-pull down of *in vivo* rG4s and the enrichment of rG4-containing transcripts. The changes in relative abundances of transcripts are then evaluated via RNA-seq. The G4RP-seq method was applied to identify *in vivo* rG4s in human by employing a cell model (53). Although the technique represents a technological advancement in ligand-based rG4 detection, the resolution of G4RP-seq is limited to the transcript level. Because the number and the location of rG4 structures per transcript cannot be determined by G4RP-seq, its results may have limited utility in rG4 genomic studies, where the accurate sequence and position of rG4s are required.

### Significance of high-throughput rG4 detection

Compared with the low-throughput and computational prediction methods, the high-throughput methods have unique strengths that are expected to bring significant improvements to the genome-wide profiling of rG4s.

First, the high-throughput methods are capable of *ab initio* detection of rG4 structures, especially for rG4s that were formed from sequences previously not considered as PQSs. A recent re-analysis of a human rG4-seq dataset published in 2016 revealed evidence for the prevalence of unusual rG4-forming sequence that fall outside of the existing rG4 structural motif definitions (98). Notable examples included two-layered rG4 structures with an extra-long loop, which could be described as a hybrid of the long-loop and two-quartet motifs, as well as RNA G-triplex motifs, which lack a fourth G-tract (98). With the expectation that more unusual rG4s in different organism models will be revealed under high-throughput methods, a better general understanding of the phenomena underlying these rG4s can be expected to emerge.

Meanwhile, when coupled with appropriate bioinformatic analysis, the high-throughput methods offer empirical evidence for rG4 detection competitive with their low-throughput counterparts. The recently proposed *rG4-seeker*, a bioinformatic program tailored for the rG4-seq method, analyses rG4-induced RTS footprints at single-nucleotide resolution and replicate-independent manner, and offers results closely resembling the outcome of a PAGE-based RTS footprinting assay (98). There are hopes that high-throughput rG4 detection methods could substitute the conventional predict-then-validate workflow for identifying novel rG4s on genes-of-interest.

Finally, other recent advancements in high-throughput methods have improved the *in vivo* detection of rG4s. The intracellular environment is known to harbour additional biophysical and biological factors that influence rG4 formation, for instance molecular crowding effects (99,100) and rG4–protein interactions (101,102) that are difficult to model *in vitro*. High-throughput methods hold promise to profile the discrepancies in the rG4 folding behaviour between *in vivo* and *in vitro* conditions, and to model the overall influence of the intracellular environment on the rG4 formation propensity.

### Current challenges in high-throughput rG4 detection

One major challenge for high-throughput methods lies in their large sample input and difficulties in profiling rG4s on low-abundance transcripts. Because these methods typically require tens to hundreds of micrograms of RNA (52), their applications are mostly limited to cultured cell or whole organism models, which can be expanded to provide the necessary RNA amount. Meanwhile, because the outcomes of rG4 detection are dependent on the number of RNA-seq reads available, the rG4 profiling results of low-abundance transcripts tend to be more ambiguous (98). Moreover, high-throughput methods frequently incorporate a poly(A) enrichment step, which compromises the success rate of rG4 discovery among the non-polyadenylated transcripts, such as non-coding RNAs, that are depleted in the process. Despite ongoing efforts to alleviate the high-input RNA requirements for these methods (103). Currently, researchers must still resort to the conventional predict-then-validate workflow when the transcript and/or model organisms-of-interest are not suitable for high-throughput detection.

Another challenge introduced by high-throughput methods lies in the subsequent analysis, which aims to reach generalized conclusions regarding biophysical features from the large numbers of rG4s detected. Conventionally, the generalizations were largely dependent on human interpretation, placing limits on both the complexity of features and the number of rG4s that can be considered in the analysis. However, rG4 formation is influenced by the complex interplay of multiple biophysical features including sequence lengths, nucleotide compositions and RNA structures in an inter-dependent manner. It remains unclear whether a human interpretation-driven approach can fully leverage the thousands of rG4s newly identified by high-throughput methods and offer new, better explanations of the phenomena surrounding the rG4 formation propensity.

A possible solution that may mitigate both limitations at once would be computational processing of high-throughput rG4 profiling data. This would involve applying analytic techniques from data science, such as machine learning, to capture the underlying properties and patterns shared among rG4s, including complex properties that are not intuitive to human interpretation. Based on the information obtained, improved rG4 prediction methods with better predictive power could then be implemented for rG4 screening in transcripts to which high-throughput methods are not well suited. From our review of existing G4 and rG4 prediction methods, we foresee several possible, but non-exclusive, pathways for such investigations:

1. To identify and model novel biophysical factors that influence the rG4 formation propensity. In the neighbouring dG4 research field, the results from high-throughput genome-wide profiling of dG4s are processed by machine learning, which enables identification of biophysical factors that are relevant to dG4 folding potentials, and the modelling of their corresponding influences on G4 formation (104). Given the similarities between dG4s and rG4s, the dG4 study model might be transferrable to rG4 research by using the profiling results of the latter in place of the former as input.
2. To automatically capture the defining properties of rG4s using an artificial neural network. A 2017 study suggested that the properties of rG4s can be automatically captured and modelled using machine learning techniques given input data consisting of non-rG4 sequences and rG4 sequences detected by high-throughput methods (Figure 2A and C). Based on that approach, the study proposed the *G4RNA screener* software, which predicts rG4s from arbitrary input nucleotide sequences (105,106). Although *G4RNA screener* can only evaluate rG4s with the canonical structural motif, it is possible that similar prediction tools that evaluate both canonical and non-canonical rG4s could be developed using a similar approach but including input data from high-throughput rG4 detection methods.
3. To establish discriminative models that infer the rG4 folding outcome of PQSs. Despite the proposal of multiple schemes to calculate the rG4 folding propensity of PQSs by numerical metrics, a successful attempt at devising a decision boundary that differentiates rG4-folding and non-rG4-folding PQSs using these metrics has yet been reported. The lack of high-quality training data containing large numbers of PQSs with correctly labelled rG4 folding/non-folding status appear to have hampered the ability of the existing discriminative modelling process to devise the required decision boundaries. Interestingly, the results from some high-throughput rG4 detection methods such as *rG4-seq* offer the possibility to label PQSs as ‘detected’ and ‘not detected’. Furthermore, a comparison of human rG4s detected/not detected in an *rG4-seq* experiment revealed differences in the distributions of two of their metrics, namely competitive secondary structure free energy and cytosine track abundance, which are well understood to associate with the rG4 folding propensity (50). This evidence suggests that the detection status of PQSs could be considered a proxy of their rG4 folding status, where high-throughput rG4 detection results may serve as suitable training data for discriminative modelling. Ultimately, the approach holds promise to establish statistical models that consider multiple rG4 structural features as input and express the rG4 folding propensity of PQSs in probabilistic terms. In such a process, high-throughput rG4 detections results would serve as both training data to construct and improve the model, and testing data for validity evaluation—similar to the approaches already used by the *cG/cC scoring scheme* (87), *G4Hunter* (88) and *G4RNA screener* (105) (Figure 2A,C).

### Approaches to connect rG4 genomics with rG4 biology

The central task in rG4 genomics is to connect the presence of rG4s to their general functions within biological systems, thus revealing, for instance, the categories of pathways and mechanisms in which rG4s play a central role, as well as the classes of proteins and other biomolecules that are designated to interact with rG4s. Although high-throughput rG4 detection methods will play a pivotal role in addressing this task by revealing more thorough information on the rG4s in genomes, it is likely that not all possible forms of rG4s will be detected by these methods, nor that all detected rG4s will be biologically relevant to the central task. Therefore, selection for functionally significant rG4s would be necessary to further approach a full understanding of rG4 genomics. For this reason, we speculate that rG4 genomics studies in the near future will be characterized by the integration of different genomics data and techniques from other disciplines of biology to identify the subsets of functional rG4s and annotate them based on the genomic functional elements that they are related to.

One viable approach could be to investigate rG4 genomics from an evolutionary biology perspective, by evaluating the conservation of rG4 sequences across multiple organisms. Given the drastic differences in the genome-wide rG4 landscape between humans (50) and other forms of life (51,96,97), any conserved rG4 structures would likely be biologically significant and could provide an entry point to identify important, conserved rG4-dependent functions.

Meanwhile, some RNA-binding proteins (RBPs), especially RNA helicases, bind rG4s on specific genes with functional implications (107–109). By comparing rG4 detection data with transcriptome-wide RNA–protein interaction probing data, for instance, cross-linking immunoprecipitation-sequencing (CLIP-seq) datasets, it might be possible to reveal the families of rG4-binding proteins and annotate rG4s based on our knowledge of their RBP interaction partners.

Finally, recent studies have suggested that some naturally occurring RNA modifications such as m6As may co-localize with rG4 structures with functional implications (110,111). Although the mechanisms underlying this co-localization have not yet been identified, recent methodological advancements in RNA modification detection (112,113) have opened new possibilities to directly compare modification loci with rG4 sequences on a transcriptome-wide scale. Further progress promises to establish new associations between rG4s and other types of modifications and could reveal connections between epitranscriptomic regulation and rG4 biology.

### Biological functions of mammalian rG4s in mRNAs and ncRNAs

**Figure 4.**
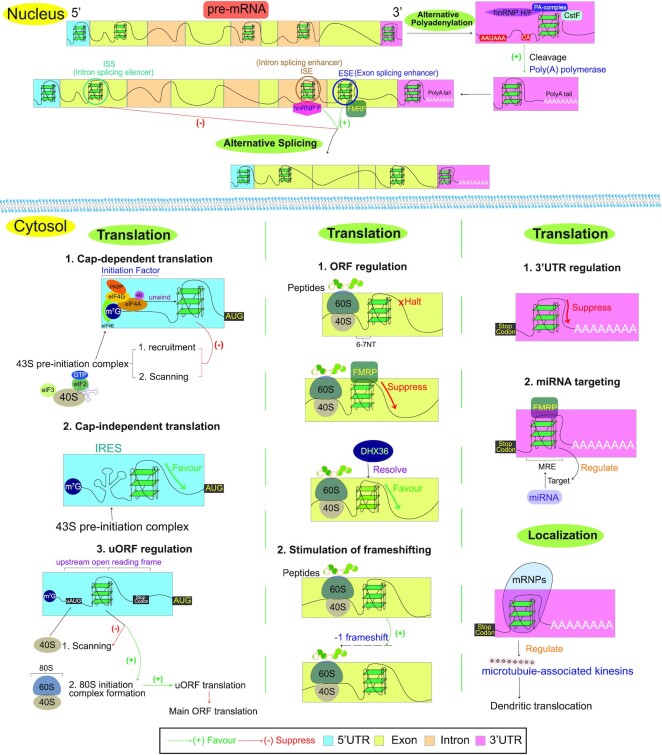
Representative roles of messenger rG4 on diverse biological processes in cells. Messenger RNAs rG4 on the 5′UTRs of mRNAs can primarily impair eukaryotic cap-dependent translation, while they promote the cap-independent translation. Meanwhile, rG4s in the uORF affect the downstream gene translation through the regulation of ribosome initiation complex formation and the ORF rG4s can regulate translational elongation and ribosomal frameshifting progression. The pre-mRNA intron rG4s in ORF can either enhance or silence RNA alternative splicing, while the pre-mRNA exon rG4s act as exon splicing enhancers. rG4s on the 3′UTR affect translation, alternative polyadenylation, alternative splicing, and RNA localization.

**Figure 5.**
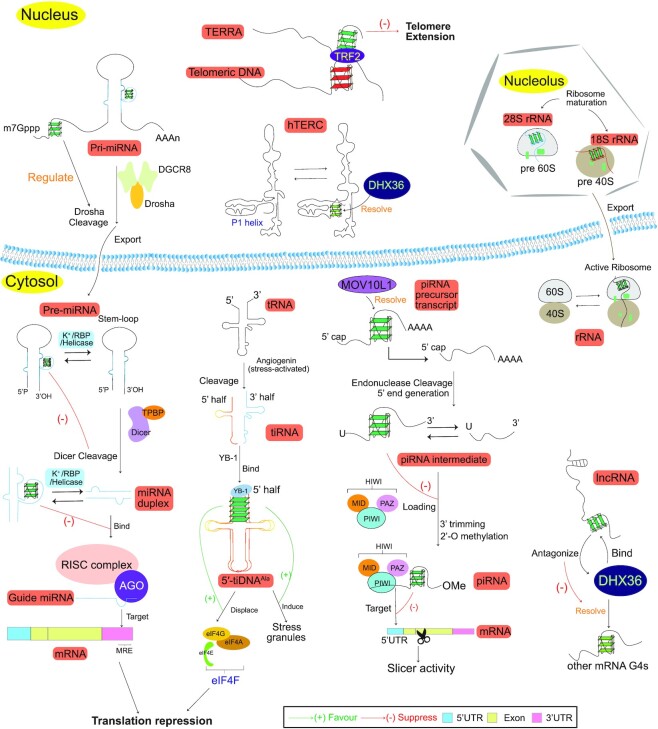
Representative roles of non-coding rG4 on myraid of biological processes in cells. rG4 in *TERRA* lncRNAs can regulate telomere length through interaction with telomere-binding protein and telomeric DNA. rG4 in *hTERC* lncRNA can be resolved by DHX36, which then facilitate P1 helix formation and its template boundary function. rG4 in lncRNA can bind to DHX36 helicase, and therefore, antagonize its unwinding activity to other RNA targets containing rG4. pri-mRNA rG4s regulate Drosha-mediated processing, pre-miRNAs rG4s inhibit DICER-mediated maturation, and miRNA rG4s abolish its loading onto RISC. rG4s can regulate the biogenesis of piRNA and lead to its inhibition of binding to the HIWI-PAZ domain as well as mRNA targeting. rG4 on the 5′ tiRNA can displace initiation factor and trigger translation repression as well as stress granules formation. The functions of rRNA rG4 are not clearly known, but may be involved in ribosomal protein recruitment and ribosome assembly.

The bioinformatic discovery of the widespread occurrence of PQSs in the human transcriptome (27), initially in mRNA, has stimulated the extensive experimental characterization of rG4 formation and function in mRNA, and over the years, numerous important roles of rG4s have been revealed (39,42,114) (Figure 4). In addition, given that the majority of the human transcriptome is composed of ncRNAs (115), recent studies have begun to report the existence and unusual role of rG4s in many classes of ncRNA such as long non-coding RNAs (lncRNAs), microRNAs (miRNAs), piwi-interacting RNAs (piRNAs), transfer RNAs (tRNAs) and ribosomal RNAs (rRNAs) (42,116) (Figure 5). Below we highlight and discuss the functions of mammalian rG4s reported in mRNAs and ncRNAs thus far.

#### 5′-UTR rG4s

Earlier studies focused on the effect of 5′-UTR rG4s on cap-dependent translation. Schaeffer *et al.* first reported the formation of an rG4 in the coding region of *FMR1* transcript, and when this specific *FMR1* rG4 motif was inserted into the 5′-UTR in a reporter gene system, it was demonstrated to suppress translation in rabbit reticulocyte lysate (41). A few years later, Balasubramanian *et al.*, using the reporter gene in rabbit reticulocytes lysate, showed that an rG4 motif naturally occurring at the 5′-UTR of the proto-oncogene *NRAS* inhibited translation (16), and their study also highlighted that the density of putative rG4s at 5′-UTR was enriched as compared with the genome average. Since then, many other 5′-UTR rG4 candidates (84,117–125) have also been reported to suppress translation in studies using either cell-free or cell-based reporter gene systems, suggesting the general repressor role of 5′-UTR rG4s in translation (Figure 4). Some notable cases exist, such as 5′-UTR rG4 in TGFβ2, in which the insertion of an rG4 motif alone in the reporter gene inhibited translation in cells, whereas the insertion of an rG4 in the context of the entire 5′-UTR of TGFβ2 augmented translation (126). Similarly, using a cell-based reporter gene, translation was suppressed by a 5′-UTR rG4 containing (CGG)_99_ repeats in *FMR1*, whereas translation was augmented by the same 5′-UTR rG4 containing only (CGG)_30_ repeats (127). Moreover, it has been suggested that the relative location of rG4s to the 5′ cap of mRNA and the thermostability of 5′-UTR rG4s can control the level of translation (128), highlighting that the neighbouring sequence context, rG4 motif density, rG4 position and stability should be considered in 5′-UTR rG4-mediated translational control.

The biochemical mechanism on 5′-UTR rG4s regulating cap-dependent translation has not been fully characterized, but a few studies have provided us an initial glimpse of this complex regulatory pathway. Eukaryotic initiation factor 4A (eIF4A) is an RNA helicase that unwinds structured 5′-UTRs to help with 43S pre-initiation complex recruitment, and a subsequent mRNA 5′-UTR scanning step is to look for the translation start codon. In one study, transcriptome-wide ribosome footprinting was performed under eIF4A inhibition with silvestrol reduced the translation efficiency of mRNAs with longer 5′-UTRs, and such mRNAs were found to be enriched in the (CGG)_4_ motif, which was later verified to form two-quartet rG4s using biophysical assays (129). These results indicated that rG4s can likely interfere with the recruitment and/or scanning of 43S pre-initiation complex, and thus with translation efficiency. This was further corroborated by a reporter gene assay under eIF4A knockdown (129), supporting an eIF4A-dependent 5′-UTR rG4-mediated translational control. More investigations are warranted to further investigate whether native transcripts containing the 5′-UTR rG4 motif function to be similarly in this system, and what other proteins, besides eIF4A, are involved in this tightly regulated process. Moreover, different 5′-UTR rG4 motifs may interplay with cis-regulatory elements such as uORF (Figure 4) (101,125), alternative competing RNA secondary structures (50,86), or recruit a distinct set of proteins (130) to modulate cap-dependent translation efficiency.

Besides cap-dependent translation, mRNA can sometimes, under stress conditions, undergo translation initiation by internal ribosome entry sites (IRESs) (131). Bonnal *et al.* conducted a deletion analysis on human fibroblast growth factor 2 (*FGF-2*) mRNA and found a 176-nt region containing two stem-loops and an rG4 motif to be a ‘structure determinant’ for the internal ribosome recruitment by IRES-containing cellular mRNAs (132). Another 5′-UTR rG4-containing transcripts, *hVEGF*, was also reported to promote IRES-mediated cap-independent translation (133) (Figure 4). The mechanistic role of rG4s in IRES-mediated translation is still unclear. Studies reported that the *hVEGF* 5′-UTR rG4 was functionally dispensable (134), and the *BAG-1* 5′-UTR rG4 was not a structural part of the IRES domain, unlike *FGF-2* and *hVEGF*, yet rG4 mutation on *BAG-1* can facilitate the reporter gene expression of both cap-dependent and cap-independent translation (135). Nevertheless, another study has shown that *hVEGF* 5′-UTR G4 can directly recruit the 40S ribosome to initiate the IRES-mediated translation (136). Further work is warranted to elucidate whether different cell types, physiological conditions and sequence contexts have a significant impact on the role of 5′-UTR rG4s in IRES-mediated translation. It may be possible to identify more 5′-UTR rG4 candidates and their functions in IRES-mediated translation by inhibiting cap-dependent translation using chemicals such as 4EGI-1 (137) or rapamycin (138), such that only cap-independent translation will be observed. In addition, it is worth considering the potential involvement of unidentified proteins that may control the equilibrium between cap-dependent and cap-independent translation processes under normal and stress conditions.

#### 3′-UTR rG4s

Similar to 5′-UTR rG4s, 3′-UTR rG4s generally act as suppressors in translational regulation (Figure 4), as illustrated in *PIM1* (139) and *APP* (140). Besides their role in translational regulation, 3′-UTR rG4s regulate other processes, such as alternative polyadenylation, miRNA targeting and mRNA localization (Figure 4).

Beaudoin *et al.* reported that the density of putative rG4s at 3′-UTRs was enriched as compared with the genome-average G4 density (141). In the same study, focusing on *LRP5* and *FXR1* transcripts bearing rG4s at the 3′-UTR, they found that 3′-UTR rG4s of both transcripts stimulated the reporter gene expression when compared with an rG4 mutant. For *LRP5*, the presence of the 3′-UTR rG4 also facilitated the efficiencies of alternative polyadenylation sites in *LRP5*, suggesting that rG4 functions as a polyadenylation regulatory element that positively modulates the use of internal polyadenylation sites. For *FXR1*, the presence of a 3′-UTR rG4 promoted the use of alternative polyadenylation sites, which resulted in the predominant generation of shorter mRNA isoforms over longer isoforms. This 3′-UTR shortening in turn impaired *FXR1*′s miRNA regulation and gene expression (141). As 3′-UTR rG4s are associated with alternative polyadenylation sites, detailed study is necessary to identify and characterize more candidate 3′-UTR rG4s that interplay with alternative polyadenylation sites and determine their impact on gene regulation.

Earlier studies reported that the 3′-UTR of *PSD* mRNA contains a G-rich region that binds to FMRP, and the G-rich region overlaps with the miR-125-binding site (142,143). Stefanovic *et al.* further demonstrated that the G-rich region can fold into thermostable rG4s and interact with FMRP, or form a double-stranded RNA conformation by hybridizing with miR-125. This alternative structure can inhibit rG4 formation and disrupt rG4–FMRP binding (144). Another study by Rouleau *et al.* reported >44 000 putative 3′-UTR rG4s to overlap with predicted miRNA-binding sites (145). Using the *FADS2* mRNA/miR-331-3p pair as an example, they showed that the formation of a *FADS2* 3′-UTR rG4 prevented the targeting of *FADS2* mRNA by miR-331-3p in a cell-based reporter gene system (145). A promising direction for further experiments would be to study the cellular factors and mechanism of 3′-UTR rG4-mediated regulation in miRNA binding and post-transcriptional regulation.

Subramanian *et al.* predicted that rG4s would be enriched in the 3′-UTR dendritic mRNAs and focused on *PSD-95* and *CaMKIIa*, two postsynaptic transcripts that were shown to be involved with the localization processes (143,146–148). Using mRNA reporters in neurons, they found that the 3′-UTR rG4 was necessary and sufficient for dendritic mRNA localization, whereas the deletion of the 3′-UTR rG4 in the reporter mRNA caused a significant reduction in mRNA translocation and also the loss of neurite signals (148). This result suggested that the 3′-UTR rG4 is a cis-acting element and can function as a zip-code for mRNA localization. Future work will be required to discover trans-acting proteins that bind with 3′-UTR rG4s in *PSD-95* and *CaMKIIa* mRNA to form the mRNA–protein complex, which facilitates dendritic mRNA transport to synapses for local translation.

#### ORF rG4s

Studies have reported that thermostable ORF rG4s can pause ribosome processing, initiate proteolysis and induce ribosomal frameshifting (FS) (Figure 4). Using a synchronized translation assay and cell-based reporter gene system, Endoh *et al.* found that rG4s in ORF can halt the translation elongation step, reporting that the ribosome stalled 6–7 nucleotides before the rG4 (149). This rG4-mediated translational elongation stalling was later found to affect the folding and proteolysis of hERalpha protein (149–151), as well as ribosomal FS events (mostly −1 type FS) in other rG4-containing transcripts in cells (150). The effect of these events was modulated by the G-runs and loop length of the rG4 involved, as well as addition of G4 ligands to the system, which can perturb the rG4 thermostability (150,152). Later, it was demonstrated that periodic fluctuation of translation suppression occurs every three nucleotides within the ORF, but not at the 5′-UTR, providing mechanistic insights on how the ribosome translocates and unfolds rG4 structures (153).

Several ORF rG4s regulate translation via their interaction with rG4-binding proteins. Over the years, FMRP has been one of the best-studied rG4-binding proteins, and the RGG motif in FMRP has been shown to be important for binding rG4s (154–156). Westmark *et al.* reported that the ORF rG4 in *APP* mRNA interacted with FMRP to inhibit its translation, without affecting mRNA stability (157). Thandapani *et al.* showed that the ORF rG4s in *MLL1* and *MLL4* mRNA interacted with the RGG motif of Aven and increased *MLL1* and *MLL4* polysomal association and thus, translation (158). In addition, two other proteins, PRMT1 and DHX36, were also reported in that study to promote *MLL1* and *MLL4* translation through Aven arginine methylation (by PRMT1) and resolving rG4s (by DHX36) (158). Interestingly, recent transcriptome-wide studies have revealed that rG4 proteins and helicases have roles in translation (101,102,159,160). It would therefore be of interest to explore whether and how, mechanistically, these proteins interact with ORF rG4s to control translation elongation, ribosomal stalling and FS.

rG4s in exons and introns regulate pre-mRNA splicing (Figure 4). With regards to exonic rG4s, Didiot *et al.* identified two rG4s in an *FMR1* mRNA-coding sequence (exon 15) and showed that they interacted with FMRP, with no effect on translation (161). Instead, these two rG4s served as exonic splicing enhancers, and bound with FMRP to regulate the *FMR1* alternative splicing and thus the level of short and long FMRP isoforms in cells (161). Fisette *et al.* demonstrated that rG4s in *BACE1* exon 3 recruit hnRNP H to control *BACE1* alternative splicing and the level of the BACE1 isoform that can proteolytically cleave APP to produce amyloid beta peptide (Aβ), which is linked to Alzheimer’s disease (162). With regards to intronic rG4s, Gomez *et al.* found an rG4 in intron 6 of the human telomerase (*hTERT*) transcript to function as an intronic splicing silencer and thereby affect the *hTERT* alternative splicing in cells, and the effect was exacerbated by titrating G4-stabilizing ligands (13). Marcel *et al.* reported that an rG4 in intron 3 of the *TP53* transcript worked as an intronic splicing enhancer to promote the splicing of intron 2, which led to differential expression of transcripts encoding distinct p53 isoforms (163). Similarly, Huang *et al.* found that an rG4 in intron 8 of *CD44* directly recruited hnRNP F and operated as an intronic splicing enhancer to regulate splicing and led to exon inclusion, which was shown to maintain the epithelial phenotype and modulate the epithelial–mesenchymal transition (EMT), an important process that drives cancer (164). Other intronic rG4 examples were also recently illustrated to regulate splicing efficiency (165–167). Future investigations may focus on whether the stability of rG4s, their proximity to RNA splicing sites, or other trans-acting factors have an impact on alternative splicing.

#### lncRNA rG4s

rG4s have been reported in telomere-associated lncRNAs (Figure 5). Telomerase is responsible for telomere maintenance, and was found to be upregulated in most cancer cells (168). Human telomerase consists of the telomerase protein (hTERT) and the telomerase RNA (*hTERC*) components, and studies have reported that the 5′-terminal of human *hTERC* contains an rG4 that interferes with the formation of a critical structural element, P1 helix, in defining the template boundary for reverse transcription (169–171). The ATP-dependent RNA helicase DHX36 was reported to bind and unwind *hTERC* rG4s in the presence of ATP (172), which enables the formation of the P1 helix structure necessary for telomerase function (173). Another telomere-associated lncRNA, telomeric repeat-containing RNA (*TERRA*), was also reported to form rG4s and be involved in the regulation of telomerase activity and DNA telomere length (174–176). *TERRA* RNA is localized to chromosome ends in the nucleus (175), suggesting a link between *TERRA* rG4s and telomere function. For example, *TERRA* rG4s can interact with telomeric dG4s to form intermolecular hybrid G4 structures to suppress telomerase activity (177). In addition, *TERRA* rG4s recruit TRF2 (178), a protein that regulates the association of *TERRA* and telomeric DNA (179). As the rG4s in both *hTERC* and *TERRA* recruit specific sets of proteins to play important roles in telomere homeostasis and genome stability, future investigations may profit from rG4 mutation/deletion and protein knockdown/knockout experiments to establish the relationship of rG4s with telomere length and genomic instability, and to assess their impact on ageing and cancer development.

For non-telomere-associated lncRNAs (Figure 5), Matsumura *et al.* reported the lncRNA FLJ39051, also referred to as G-quadruplex-forming sequence containing lncRNA (*GSEC*), to be localized in the cytoplasm and upregulated in colorectal cancer cells (55). *GSEC* is a 753-nt-long lncRNA, in which an rG4 structure was detected at nucleotide positions 11–26. Using multiple assays, the rG4 in *GSEC* was shown to interact directly and specifically with DHX36, and antagonized the function of DHX36 by acting as a molecular decoy to block DHX36 from interacting with its target RNAs, such as the 3′-UTR of *PITX1* (55). Mutational and cellular analysis further verified that *GSEC* promoted the motility of colon cancer cells by suppressing the function of DHX36 via the rG4 structure in *GSEC* (55). It is currently unclear how many lncRNA rG4s there are, and future studies may discover novel lncRNA rG4–protein interactions, as well as their regulatory mechanism *in vivo*.

#### miRNA rG4s

rG4s play roles in every step of the miRNA biogenesis and function (Figure 5). For primary miRNA (pri-miRNA), Rouleau *et al.* predicted >9% of human pri-miRNAs contains rG4s (180). They identified rG4s near the Drosha cleavage site in human tumour suppressor pri-miRNAs such as pri-miR200c, pri-miR451a and pri-miR497, and reported that their formation *in vitro* was favoured in the presence of PhenDC3 (180). To highlight the pri-miRNA rG4s’ gene regulatory roles in cells, they performed mutation analysis of the effect of rG4s on mature miRNA production (180). For pri-miR497, they found that disruption of the rG4s caused a decrease in the mature miRNA level, indicating the rG4 motifs could contribute to the regulation of pri-mRNA processing. However, this effect varied depending on the rG4 location and sequence context, as pri-miR451a showed an increase in the mature miRNA level when rG4 was mutated (180). Considering the tumour-related regulatory role of the above-mentioned miRNAs, the rG4 on pri-miRNA could be further investigated as a potential target for cancer therapeutics.

Several key studies have demonstrated that rG4s form in precursor miRNAs (pre-miRNAs) (71,181,182), and are in competition with the RNA stem-loop conformation. The stabilization of rG4 or destabilization of the stem-loop conformation in these pre-miRNAs by potassium ions, G4 ligands or lock nucleic acids (LNAs) was shown to inhibit Dicer recognition and processing, which in turn affected the mature miRNA production and downstream gene regulation (71,181-183). In addition, two studies found that the expression of mature miRNAs could be restored by treatment with TmPyP4, an rG4-destabilizing ligand, to unfold rG4 (182,184). Recently, it has been reported that a single-nucleotide polymorphism (185), an RBP (186), and a G4 helicase (187) were involved in shifting the equilibrium between the rG4 and stem-loop conformations, providing plausible biochemical mechanisms for further investigation.

For mature miRNA, initial studies primarily focused on the *in vitro* biophysical characterization of rG4 formation in mature miRNAs (188–190). In 2018, Chan *et al.* predicted >100 rG4s in human miRNAs, and focused on one of the candidates, miR765, which is associated with liver, prostate and bone cancers (56). They identified and verified the formation of the rG4 in miR-765 *in vitro*, and further performed reporter gene assay in cells to illustrate the role of the miRNA rG4 in gene regulation, showing that its function can be amplified by the addition of the rG4-stabilizing ligand NMM (56). Other examples of mature miRNA rG4s have also been reported (56). However, like for pre-miRNA rG4s, these experiments were performed using reporter genes. Therefore, their regulatory role over native mRNA targets and the effect on resultant protein products remain to be elucidated.

#### piRNA rG4s

rG4s also play roles in piRNA biogenesis and function (Figure 5). For piRNA precursor transcripts, Vourekas *et al.* first analysed the CLIP data of MOV10L1, an RNA helicase important in piRNA biogenesis, and identified a significant enrichment in piRNA clusters containing more Gs in the bound areas (191). Using the G4-specific antibody BG4 in testis lysate, they reported an increased level of rG4 formation within piRNA precursor transcripts in a MOV10L1 knockout as compared with wildtype mice, suggesting that MOV10L1 interacted with and resolved rG4s (191). This rG4-specific binding and unwinding activity of MOV10L1 was recently validated (192). The overall effect of MOV10L1 helicase activity on RNA secondary structures such as rG4s is to unwind them to single-stranded piRNA precursor transcripts, enabling them to be cleaved by the endonuclease. For mature piRNA, Balaratnam *et al.* predicted a 5.5% rate of rG4-forming sequences in human piRNA (193). They demonstrated, using biophysical assays and RNase T1 structure mapping, that piR-48164 can form an rG4 (193). It was then shown, using a reporter gene system, that the formation of rG4 in piRNA prevented its binding to the PAZ domain of HIWI proteins and base-paring to mRNA targets, which in turn inhibited target gene silencing in cells (193). This implies that piRNA rG4s can affect gene silencing, and it is of interest to test these effects in native transcripts and to determine the molecular basis of this piRNA rG4-mediated regulatory mechanism.

#### tRNA rG4s

rG4s have been reported in tRNA-derived stress-induced RNAs (tiRNAs) (Figure 5). Ivanova first reported that the 5′-terminal oligoguanine (TOG) motif in 5′-tiRNA^Ala^ and 5′-tiRNA^Cys^ folded into an intermolecular rG4 in tiRNAs, and interacted with the cold shock domain (CSD) of translational repressor YB1 protein (194). Lyons *et al.* further revealed that the tetramolecular rG4 structure consisted of five G-tetrads formed by the TOG motif of four tiRNA^Ala^ copies, and the rG4 was in equilibrium with a hairpin structure (195). The biological relevance of this rG4 was then studied by using 7-deazaguanine (7-deazaG) to replace the guanine in 5′-tiRNA^Ala^, and comparing the resulting construct with the wild-type 5′-tiRNA^Ala^. Biochemical and functional analyses demonstrated that the wild-type 5′-tiRNA^Ala^, but not the 7-deazaG-substituted 5′-tiRNA^Ala^, interacted with YB1 protein, displaced the eIF4F complexes and triggered the formation of stress granules, which then led to the inhibition of translation (195). These results demonstrated that rG4s are important for tiRNA bioactivity, and as tiRNAs are stress-induced, the next step will be to examine the rG4s’ role in diverse stress conditions and diseases.

#### rRNA rG4s

The study of rG4s in rRNA was very limited until recently (Figure 5). Mestre-Fos *et al.* first identified G-tracts in expansion segment (ES) tentacles in rRNA large ribosomal subunits (LSUs), and found them to be conserved in chordates. They verified the thermostable rG4 formation in ES7 and ES27 *in vitro*, and further identified RNA-binding proteins such as FIP1, FUS, DDX3 and hnRNP H, some of which had been previously reported as rG4-binding proteins (196). The same group also later reported additional rG4s on the surfaces of small ribosomal subunits (SSUs) *in vitro*, such as es3 and es6, which suggested that both the LSUs and the SSUs of the human ribosome contain multiple rG4s (197), which may assist in ribosome assembly or associated protein recruitment. One recent study showed that these rRNA rG4s form in cells and can control heme bioavailability (198). It is of interest to explore whether future RNA structure mapping or 3D structure determination techniques can capture a folded rG4 state in the ribosome, or the binding of an rG4 with associated ribosomal proteins *in vivo*.

### Biological functions of viral, plant and bacterial rG4s

Besides being reported in mammalian systems as described above, rG4s also form and have functions in other species, including but not limited to viruses, plants and bacteria. The existence and roles of viral G4s have been excellently reviewed elsewhere recently (199,200), and therefore we highlight some representative viral rG4 examples and then focus on SARS-CoV-2 below. The study of rG4s in plants and bacteria, however, is still in its infancy, and below we highlight key findings from recent years and compare them to the results in mammalian systems.

#### rG4s in viruses

The prediction of PQSs in the genome of all known DNA and RNA viruses that can infect humans has been comprehensively performed recently, and the analysis showed that the occurrence and location of PQSs in the viral genome are orderly arranged and features characteristic of each virus family and species (57). Over the years, experimental studies have been carried out to verify the viral rG4 formation, to identify viral rG4 binding proteins and to develop antiviral G4 ligands (199,200). For DNA viruses, Epstein–Barr virus (EBV)-encoded protein EBV nuclear antigen 1 (EBNA1) was reported to be bind to rG4s through its linking region 1 (LR1) and LR2 (201). Besides, the EBNA1 mRNA can form rG4 itself and take part in the cis-acting regulation of viral mRNA translation (202). Similar to EBNA1, the mRNA of latency-associated nuclear antigen (LANA) in Kaposi’s sarcoma-associated herpes virus (KSHV) was found to form rG4, which inhibited the translation of LANA (203). Recently, an rG4 named PQS18-1 in the 3′UTR of *IE180* gene in Pseudorabies virus (PRV) was reported to form *in vivo* and regulates the replication of PRV by enhancing the expression of IE180 (204). For RNA viruses, conserved rG4s have been predicted in the genomes and later experimentally validated in both positively strand virus like Zika, hepatitis C virus (HCV) and the (SARS-CoV) and negatively stranded virus like Ebola virus (205–207). In addition, rG4 ligands were reported to interefere with the gene activity of different RNA viruses (205–208). Similarly, two highly conserved rG4s that are located in the Nipah virus G and L genes respectively were recently demonstrated to interact with TMPyP4 (209). Besides that, the nucleocapsid protein (NCp7) from human immunodeficiency virus type 1 (HIV-1) was found as the first viral protein to unwind rG4s for the reverse transcription to proceed, and the effect was counteracted by BRACO-19 G4 ligand (210).

Severe acute respiratory syndrome coronavirus (SARS-CoV) is a highly contagious human viruses which was identified in 2003 (211). Since 2019, a novel enveloped RNA betacoronavirus named SARS-CoV-2 which caused coronavirus pneumonia (COVID-19) has become a severe threat to global public health (212), causing >2.6 million human death and >121 million infected so far. Comparing to SARS-CoV, fewer rG4s were predicted in SARS-CoV-2 (213). In the RNA genome of SARS-CoV-2, about 25 PQSs were found and localized in the open reading frames of ORF1 ab, spike (S), ORF3a, membrane (M) and nucleocapsid (N) genes (214,215). Two rG4s from ORF1ab and S respectively were found to interact with viral helicase nsp13, which play a role in facilitating the replication and transcription of the SARS-CoV-2 (214). Moreover, an rG4 named RG-1, which locates in the CDS region of SARS-CoV-2 nucleocapsid phosphoprotein (N) has been demonstrated to form in live cells, and can be further stabilized by G4 ligand PDS derivative (PDP). The expression of SARS-CoV-2 N were decreased both *in vitro* and *in vivo* by PDP treatment, which indicates that rG4 in SARS-CoV-2 may be a novel target for developing antiviral drugs against COVID-19 (216). It was reported that the SARS-unique domain (SUD), which is thought to be related to its pathogenicity in the SARS-CoV showed binding preference to rG4s (217). Recently, Zhang *et al.* found that SARS-CoV-2 possesses a similar SUD domain which has eight key conserved amino acid in numerous SARS-CoV-2 samples across the world. It indicates that the SARS-CoV-2 may have similar mechanism to SARS-CoV in gene regulation, and the interaction between rG4 and rG4 binding protein domain, e.g. SUD domain of nsp3, possibly leads to dimerization and the instability of transcription or the translation efficiency (218,219). Cumulated evidences have showed that rG4s have involved in various biological processes in viral system like transcription, reverse transcription, replication and translation, highlighting that rG4s are promising targets for antiviral drug development. The next step is to develop individual rG4-specific tools for precise viral rG4 targeting.

#### rG4s in plants

In *A. thaliana*, rG4s were predicted to be over-represented in protein-coding genes, and under-represented in ncRNAs such as rRNAs and tRNAs (220). In addition, the same study predicted >400 putative 5′-UTR rG4s in the transcriptome of *A. thaliana* (220). Kwok *et al.* conducted structural analysis and functional characterization of an rG4 located in the 5′-UTR of *ATR* mRNA in *A. thaliana*, and it was shown using a cell-based reporter gene system that the rG4 played an inhibitory role in translation (221). Comparative sequence analysis of the *ATR* transcript indicated that the rG4 motif was conserved among 14 plant species, and biophysical assays verified that these rG4s folded into thermostable forms (221). Notably, the translational suppression role of the *ATR* 5′-UTR rG4 in *A. thaliana* resembled that reported for most 5′-UTR rG4s in mammalian systems (discussed above), suggesting that similar molecular mechanisms may apply to control 5′-UTR rG4-mediated translation processes across plants and mammals.

Cho *et al.* performed a comparative transcriptome analysis of the phloem–cambium region of three plant species and identified an uncharacterized zinc-finger (ZnF) protein referred to as JULG1. It was shown to specifically bind and induce a 5′-UTR rG4 of SMXL4 and SMXL5, which are key regulators of phloem formation, and suppress their translation. Reduced production of JULG1 restricted phloem differentiation and strikingly increased sink strength per seed (222). Notably, the mechanism underlying post-transcriptional regulation of phloem differentiation is exclusively conserved in vascular plants (222), suggesting that this rG4 and its binding protein can influence the plant development through post-transcriptional regulation. Interestingly, ZnF proteins have also been reported to bind to G4s in humans. For example, the ZnF protein CNBP/ZNF9 specifically binds to G-rich regions in the target mRNAs and promotes their translation, potentially by resolving rG4 structures in the target mRNAs (159). Taken together, these results show that ZnF-containing proteins could directly interact with G4s and may have diverse roles in G4-associated gene regulation in plant and mammalian species.

More recently, Yang *et al.* carried out transcriptome-wide mapping of rG4s in plants and identified hundreds of rG4s *in vivo* (97). In agreement with computational prediction, *in vivo* rG4 mapping data showed that two-quartet rG4s were identified more frequently than three-quartet rG4s, and in the coding region, the rG4s were enriched relative to UTRs (97). The results were largely consistent between *A. thaliana* and *O. sativa* (97). The authors further characterized one of the 3′-UTR rG4 candidates, *HIRD11*, which encodes a KS-type dehydrin, and demonstrated that the rG4 has an inhibitory role in translation. Using a plant root growth assay, the same study also showed a significant difference in phenotype between the candidate 3′-UTR rG4 and an rG4 mutant (97). In contrast to the earlier report by Guo *et al.* of the global unfolding of rG4s in mice, humans and yeast (51), rG4s in plants show a greater tendency to be folded *in vivo*, highlighting that the physiological environment may be quite different between plants and mammalian species. It is also possible that rG4s only fold in specific cell types or environments in mammalian systems. As future subjects of study, we propose dissecting the biochemical mechanisms that underlie the above-mentioned phenomena and identifying any novel rG4-binding proteins in plants, as well as performing transcriptome-wide rG4 mapping under diverse cellular conditions across different species.

#### rG4s in bacteria

Genome-wide predictions of PQSs have been performed in the genomes of *Escherichia coli* (223), *Deinococcus radiodurans* (224), *Xanthomonas* sp., *Nostoc* sp. (225) and *Mycobacterium tuberculosis* (226), and experimental-based DNA G4-seq was performed in *E. coli* to identify dG4s *in vitro* (227). In 2016, Guo *et al.* reported that rG4s were depleted in bacteria, and proposed that unlike in mammalian cells, which have evolved mechanisms to deal with rG4s (e.g. rG4-unwinding helicases and single-stranded G-rich RNA-binding proteins), rG4s may be evolutionarily selected against in bacteria (51). In addition, they demonstrated that artificial insertion of an rG4 into a bacterial reporter gene system caused a reduction in bacterial growth, as well as aberrant translation products (51).

Recently, Shao *et al.* used QUMA-1, an rG4-specific fluorescent probe, to demonstrate the presence of rG4s *in vitro* and *in vivo* in 10 diverse model bacterial species (96). Moreover, through rG4-seq, they obtained 168 and 161 *in vitro* rG4 sites in *E. coli* and *Pseudomonas Aeruginosa*, respectively, which were all distributed in the CDS regions (96). Using biophysical assays and a cell-based reporter gene system, they verified the formation of rG4 in the coding region of *hemL*, a metabolic gene, and demonstrated an rG4-dependent regulatory system in *E. coli* (96). Similarly, they revealed the function of rG4 in the coding region of *bswR*, a virulence gene, in *P. aeruginosa*, and illustrated by mutagenesis and phenotypic assays that the presence of rG4 affected the bacterial flagella and biofilm formation of *P. aeruginosa* by upregulating the expression of *bswR* (96).

Research thus far into bacterial rG4s (51,96,228,229), reveals that they display both positive and negative regulatory roles in translation. This difference may be dependent on rG4 stability, rG4 position and the flanking sequence context of the transcript-of-interest. Currently, studies of naturally occurring rG4s in bacteria are very limited. Further research is warranted to identify new bacterial rG4s using the tools developed in the mammalian and plant systems mentioned above, and explore and characterize their functions in different bacterial strains and stress conditions, which will help establish the general roles and effects of rG4s in bacterial systems, and allow a comprehensive comparison with the mammalian, viral and plant systems.

### Current challenges and future perspectives of studying rG4 biological functions

Our understanding of the function of rG4s has expanded to numerous classes of RNA and diverse living organisms (Table 1). However, challenges remain with regard to establishing the relationship of rG4 motifs with other cis-regulatory elements on RNA to fine-tune gene activity, revealing the direct interactions of rG4s with the plethora of biomolecules that prevail in cellular milieu, elucidating the biological mechanisms and consequences of many rG4-mediated cellular processes, and developing rG4-specific tools to selectively target functionally and pathologically important rG4s for different applications.

**Table 1.** Representative rG4 functions and examples reported to date

Region	Representative functions	Key examples	References
5′UTR rG4	Suppress or promote translation	*FMR1, NRAS, Zic-1, ESRα, CCND3, TRF2, ADAM10, MT3, BCL-2, TGFβ2, FGF-2, hVEGF, BAG-1, ATR, SMXL4/5*	(16,41,117–121, 123,124,126,127, 132–136,221, 222)
3′UTR rG4	Suppress translation, regulate alternative polyadenylation, interfere with miRNA targeting, control mRNA localization	*PIM1, APP, HIRD11, LRP5, FXR1, FADS2, PSD-95, CaMKIIa*	(97,139–141, 145,148)
ORF rG4	Regulate translation, control alternative RNA splicing	*APP, MLL1, MLL4, hemL, bswR, hTERT, FMR1, BACE1, TP53, CD44*	(13,96,157,158, 163,164)
lncRNA rG4	Regulate telomerase activity, telomere homeostasis and genome stability, antagonize DHX36 helicase function	*hTERC, TERRA, GSEC*	(55,172,177–179)
miRNA rG4	Regulate miRNA maturation and post-transcriptional regulation, control miRNA targeting	*pri-mir200c, pri-mir451a, pri-mir497, pre-miR92b, pre-let7e, pre-miR149, miR765*	(56,71,145,181, 182)
piRNA rG4	Regulate piRNA metabolism and control piRNA targeting	*piR-48164*	(193)
tRNA rG4	Inhibit translation	*5′-tiRNA^Ala^, 5*′*-tiRNA^Cys^*	(194,195)
rRNA rG4	Mediate ribosome assembly and associated protein recruitment	*ES7, ES27, es3, es6*	(196,197)

Regarding the interplay between rG4s and cis-regulatory elements, several pilot studies have illustrated that rG4 formation can control the accessibility of cis-regulatory elements on a transcript-specific basis (141,144). In addition, transcriptome-wide rG4 mapping studies have shown strong positional correlations between rG4s and polyadenylation signals and miRNA target sites in mRNA (50), suggesting that rG4s and these cis-regulatory elements might coordinate to regulate RNA metabolism and gene expression on a global scale. It remains to be deciphered whether such dynamic regulation is linked to specific cellular status and cell type. Moreover, given that there are hundreds of naturally occurring RNA epigenetic marks (230), several of which are on guanines (e.g. internal m7G), their impact on rG4 formation and biological role in cells will be worthy topics for future investigation. One initial study has shown that internal m7G in pre-miRNA precludes rG4 formation, resulting in mature miRNA regulation (231).

Regarding the rG4 interactors in cells, a dozen G4-binding proteins have been identified over the years (38,232); however, our current understanding of the protein sequence and structural requirements for rG4 binding is still limited, making it challenging to fully appreciate the complex network mediated by rG4–protein interactions. The RGG and ZnF motifs have been identified in many G4-binding proteins (233,234), and lately, a new protein motif was also reported to interact with G4 specifically (235). With accumulating information about G4-binding proteins as well as the better availability of high-resolution G4–protein complexes, it may be possible to predict new rG4-binding proteins using primarily protein sequence/structure information. Moreover, with the growing list of cell-based RNA-centric methods to detect transcriptome-wide RNA–protein interactions (236,237), we anticipate that with some degree of adaptation, these approaches can be readily applied to reveal rG4–protein interactions *in vivo*, which will greatly expand our repertoire of known rG4-binding proteins. Likewise, many existing techniques for detecting RNA–RNA and RNA–chromatin interactions (236,237) can also likely be repurposed for the detection of potential rG4–RNA and rG4–chromatin interactions. Besides those biomolecules mentioned above, rG4s have also been reported to interact with small molecules in cells, such as heme (198,238) and polyamines (239,240), which necessitates new approaches to identify rG4-interacting small molecules/metabolites *in vivo*.

Regarding the biological impact of rG4s and/or rG4-binding partners in cellular processes, reports have uncovered novel functions of rG4s that warrant further in-depth investigation. First, rG4s can facilitate RNA accumulation and phase separation in human and plant cells (241,242), and it will be interesting to see if this has any link with the assembly and function of subcellular compartments, such as stress granules and p-bodies, and under what conditions these membraneless organelles and processes can be regulated, for example by the expression level of rG4 helicases (102). Second, rG4-binding proteins and G-rich-binding proteins such as DHX36 and hnRNP F/H mediate translation by resolving rG4s and keeping them in single-stranded G-rich conformations, and this was linked to genomic instability and therapy resistance in glioblastoma (160). Elsewhere, in mice, DHX36 has been reported to unwind rG4s in pre-miRNA26a to facilitate mature miRNA26a production under healthy conditions but not in obese mice (187). This rG4-mediated regulation was shown to contribute to hepatic insulin resistance and the dysregulation of liver metabolism (187). Future studies may also focus on the biological consequence of rG4s or rG4-binding partners in other diseases and physiological changes, such as ageing, cell differentiation and cell development (243). Last, rG4s have been found in approximately 30% of dendritic transcripts, such as CaMKIIa and PSD95, which are localized in cortical neurites (148). Moreover, several key neuronal proteins are G4-specific, such as FMRP (156) and FUS (244), implying that rG4s may be linked to gene regulation and function in neurological diseases.

Regarding rG4-targeting tools, several hundred G4-selective ligands have been developed so far (245); however, given the structural similarity of dG4s and rG4s, only a few ligands have been reported to possess some degree of specificity towards rG4s, such as cPDS (36) and QUMA-1 (54). Few studies have coupled chemically functionalized or anchoring guanine with antisense oligonucleotides or peptides to achieve greater target specificity (246–248). New approaches will be needed to enable the selective targeting of rG4 classes (over dG4s and non-G4s) or even of individual rG4s for different applications. Recently, two L-RNA aptamers were developed to target rG4 structure, one of which prefers to bind generally to many rG4s (249), whereas the other prefers to bind specifically to individual rG4s (250), suggesting that the development of universal rG4 and selective rG4 binders is feasible. For these above-mentioned approaches, the cell permeability and associated cellular cytotoxicity need to be studied in more detail across different cell lines and species in the future. In addition, their specific target binding in native intracellular transcript contexts and their ability to interfere with different biological processes need to be verified to illustrate their utility. As rG4s have been associated with diseases (42,116,251), rG4-targeting tools will help decipher and manipulate the biochemical mechanism of these rG4-associated gene regulations and functions.

## CONCLUSION

The study of rG4 has progressed significantly over the past few years, and the advancement in technology has naturally led to important new methods and basic discoveries in rG4 biology. We anticipate that the next key challenge will be to further investigate where, when and how rG4s are being formed and dynamically controlled by cis- and/or trans-regulatory elements in cells, as well as the specific underlying rG4-mediated biochemical mechanism and functional consequences in different organisms (see future perspective sections above). Collectively, these fundamental insights will facilitate us to explore the origin and evolution of rG4s, and enable us to decipher the missing link between rG4s and diseases. The outcomes of these will undoubtedly be highly valuable in the design and development of tools to target rG4s and rG4-associated pathways for diverse biological, biomedical and biotechnological applications. We look forward with enthusiasm to new suite of methodologies and biological breakthroughs to be unraveled in the near future.
